# Retime-mapping terahertz vernier biosensor for boosting sensitivity based on self-reference waveguide interferometers

**DOI:** 10.1016/j.fmre.2024.12.002

**Published:** 2024-12-14

**Authors:** Liang Ma, Fei Fan, Weinan Shi, Yunyun Ji, Xianghui Wang, Shengjiang Chang

**Affiliations:** a Institute of Modern Optics, Nankai University, Tianjin Key Laboratory of Micro-scale Optical Information Science and Technology, Tianjin 300350, China; b Tianjin Key Laboratory of Optoelectronic Sensor and Sensing Network Technology, Tianjin 300350, China

**Keywords:** Terahertz, Waveguide interferometer, Vernier biosensor, Self-reference detection, Sensitivity enhancement

## Abstract

The optical vernier effect serves as a potent mechanism for boosting sensitivity and accuracy in the communication band, which is a prominent hotspot in coherent detection. Extending vernier gain to the terahertz window exhibits significant appeal in next-generation wireless communication and high-resolution sensing. Here, a terahertz vernier biosensor is constructed utilizing two overlapping Mach-Zehnder interferometers within a three-channel metallic waveguide. The self-reference feature of the vernier biosensor facilitates a sensitive envelope, and the vernier gain significantly amplifies the detection sensitivity and accuracy from the superposition of slightly detuned terahertz interference spectra mapping within the time-frequency-time domain. An exalting sensitivity of 22.54 THz/RIU is demonstrated at operating frequencies near 0.9 THz and experimentally shows immense sensing performance in detection sensitivity and accuracy of biochemical sample areic mass are 10^7^ GHz/(g/mm^2^) and 10^−8^ g/mm^2^, respectively, presenting an enhancement of > 3000% compared to a single interferometer. Moreover, the sensor is employed to assess the amino acid oxidation characteristic curve analysis in the terahertz range, which assists in identifying specific amino acids. The validation of the vernier effect operating in the terahertz regime demonstrates the development of a rapid and label-free assistance tool for the identification of biochemical samples.

## Graphical abstract

**Figure:**
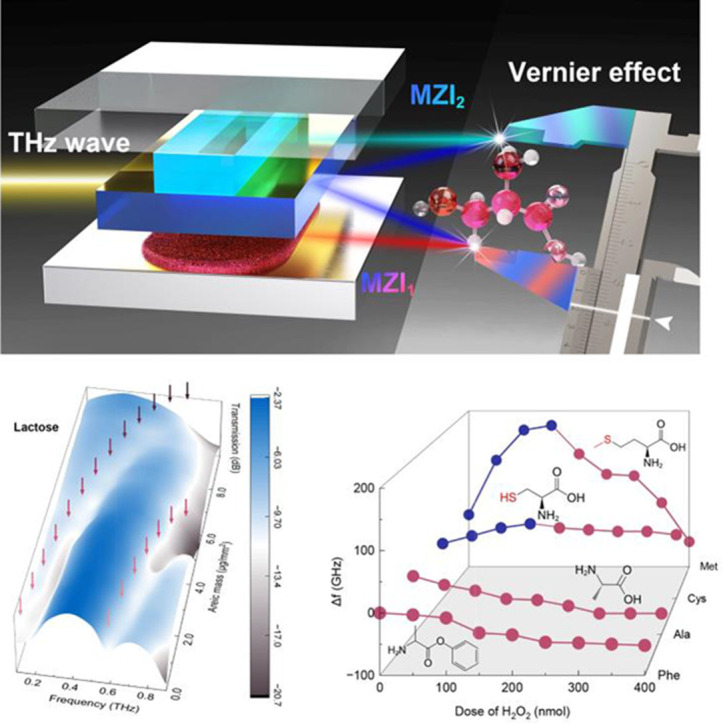

## 1 Introduction

Coherence detection incorporating optical vernier modulation (i.e., through the introduction of reference interference) [1], as a fundamental mechanism for optical measurements, has achieved remarkable breakthroughs in communication [2], imaging [3], measurements [4], and sensing [5]. The utilization of an optical vernier in coherent detection significantly enhances sensitivity and accuracy in the spectrum [6,7], akin to a vernier caliper that amplifies precision in length measurement. Optical verniers typically rely on the superposition of two interference signals with slightly detuned free spectral ranges (FSRs): one signal corresponds to the main scale of a vernier caliper, while another resembles the vernier scale. Their overlapping interference spectra generate a highly sensitive envelope. The vernier modulation mechanism is extensively employed in designing vernier filters [8], lasers [9], and sensors [10]. Despite significant advancements in coherence detection and vernier effect within visible and infrared bands [11,12], their applications remain limited in the terahertz (THz, 10^11^–10^13^ Hz) regime. Considering that the THz range is an essential window for biochemical sensing [[13], [14], [15], [16]], molecular spectroscopy [17,18], and next-generation wireless communications [[19], [20], [21], [22], [23]], extending coherent detection and optical vernier gain to this region is very attractive. However, constraints imposed by waveguide structures, coupling modes, and operational frequency range often present obstacles to advancement in this band.

The research on THz biochemical sensing based on metasurfaces has achieved significant breakthroughs, demonstrating the successful integration of metasurfaces with functional materials and deep learning to address the intricate challenges associated with trace detection [[24], [25], [26]]. To further enhance the light-matter interactions, waveguide-based sensing solutions are anticipated to emerge as a novel approach. For example, the recent THz topological waveguides have emerged as a promising solution for highly efficient coupling at sharp bends, showcasing significant advantages [27]. Simultaneously, their high integration capability, low-loss characteristics, high-speed transmission, and flexible structural size make them achieve remarkable on-chip communication and sensing breakthroughs [[28], [29], [30]]. However, their operational range is typically confined to a narrow frequency band of <100 GHz, owing to the constraints imposed by their structural dimensions. In sensing applications, the limited sensitivity is primarily attributed to the weak interaction between the electromagnetic wave and the sample outside the transmission channel. Furthermore, implementing such waveguides necessitates intricate structural designs and costly machining processes. These factors present challenges to advancing coherence detection with optical vernier in such a waveguide. THz metallic parallel-plate waveguide (PPWG) presents a productive solution for simplifying the waveguide structure, enriching operating bands, and enhancing detection sensitivity [31]. Traditional PPWG-based modulators and sensors are commonly integrated with microstructures, such as leaky-wave antennas [32,33], resonant photonic cavities [34], Bragg gratings [35], and photonic crystals [36]. However, challenges like limited sensitivity and restricted operational frequency often arise. Waveguide interferometers are anticipated to serve as a pivotal structure for addressing this issue, aiming to boost the sensitivity and overcome operational frequency limitations. Therefore, investigating the behavior of waveguide interferometers with the vernier effect in the THz band holds significant importance.

Here, we have demonstrated the feasibility of a self-referential THz vernier biosensor, providing the experimental validation of waveguide interferometers with the vernier gain in the THz band. The two overlapping Mach-Zehnder interferometers (MZIs) are creatively constructed to form a self-referential THz vernier biosensor by employing a multi-layer design to establish three transmission channels within the PPWG. The transmitted signal is continuously mapped through Fourier transforms twice into the time-frequency-time domain to modulate and demodulate the coherent signal. Theoretical analysis and transmission experiments indicate that the vernier sensor exhibits an exceptionally sensitive spectral response to variations in both structural and dielectric parameters. Consequently, the detection sensitivity and accuracy are boosted by over 3000% in sensing applications with the gain of the vernier effect. More significantly, the exceptional performance enables the detection of amino acid oxidation characteristic curves in the THz range, which facilitates the identification of specific amino acid types based on significant differences.

## 2 Materials and methods

### 2.1 Simulation

The electric field distribution in Fig. 4e and the simulation results in Supplementary Information were calculated using commercial Ansys Optics software based on the Finite Difference Time Domain (FDTD) solver. The perfect electrical conductor (PEC) conditions were applied to simulate metallic waveguides, and the complex refractive index models were used to simulate biaxially oriented polypropylene (BOPP) material (a refractive index *n* = 1.5 and an extinction coefficient *κ* = 0.005) and SiO_2_ (*n* = 2.05 and *κ* = 0.005).

### 2.2 THz experimental system

**Fig. 1:**
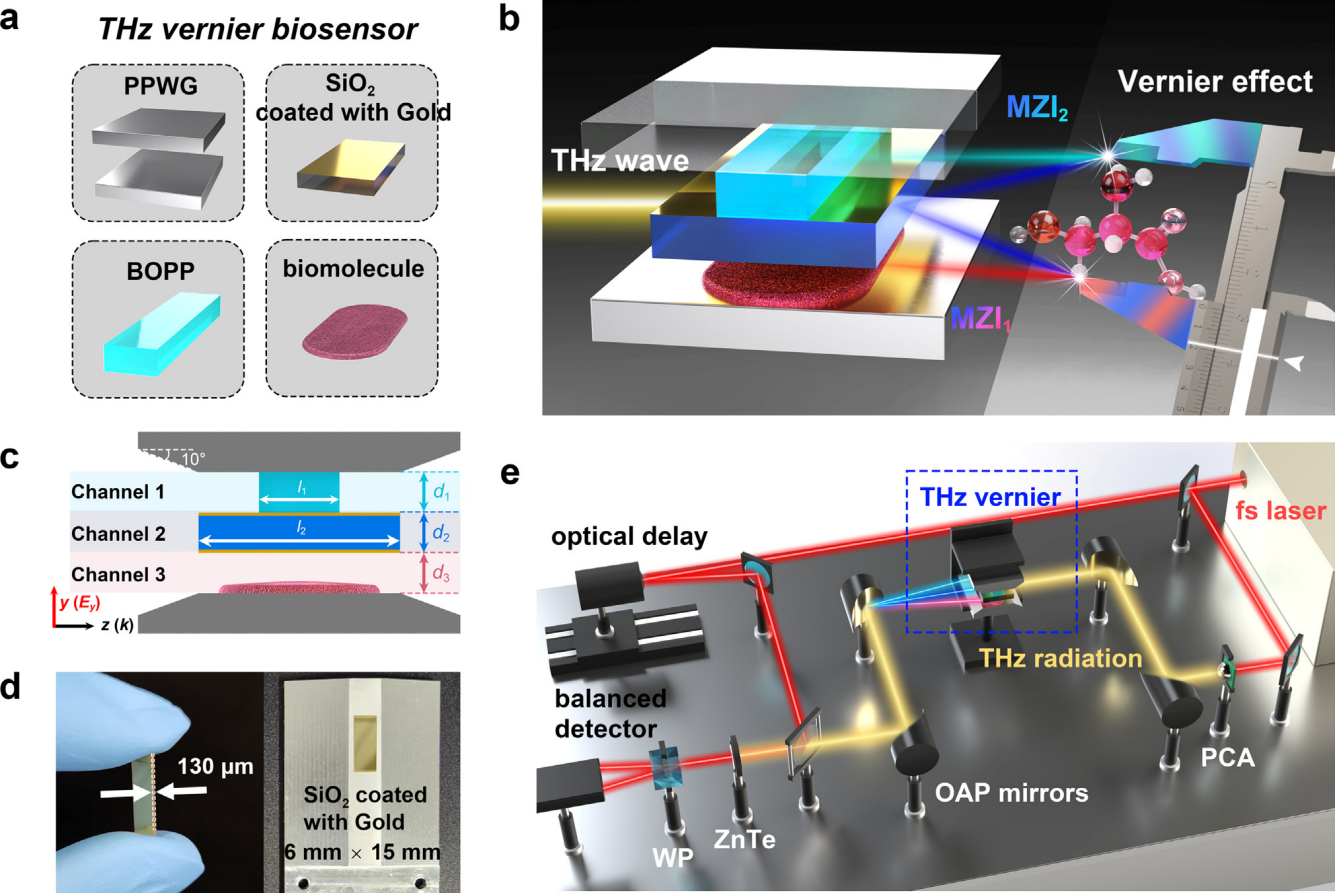
**Principle and design for THz vernier biosensor.** (a) The fundamental components and (b) operational principles of THz vernier sensor. (c) Two-dimensional diagram of three-channel overlapping MZIs structure. Light blue, dark blue, and light red backgrounds represent Channels 1–3. (d) Photo of SiO_2_ coated with gold films (left) and front view of THz vernier biosensor (right). (e) THz-TDS system in experiments. PPWG, parallel-plate waveguide; BOPP, biaxially-oriented polypropylene; MZI, Mach-Zehnder interferometer; WP, Wollaston prism; OAP, off-axis parabolic; PCA, photoconductive antenna; fs, femtosecond. (For interpretation of the references to colour in this figure legend, the reader is referred to the web version of this article.)

Fig. 1e illustrates the THz-TDS system. Firstly, a femtosecond (fs) laser (780 nm) is split into two beams: one is focused on the photoconductive antenna (PCA) as a pumping source to excite THz radiation, while another is utilized for coherent detection. Subsequently, the PCA (50 µm LT-GaAs antenna) functions as a prominent generator of wideband linearly polarized THz radiation. When the fs laser from the detection path and the THz wave converge into the ZnTe crystal, changes in birefringence within the ZnTe are induced. The polarization energy of beams split by the Wollaston prism varies depending on THz wave intensity, and the differential signal is quantified using a balanced detector. Adjusting the time delay between THz waves and the fs laser can measure both the amplitude and phase of the output THz wave.

During experiments, the THz vernier biosensor is positioned at the focal center of the THz radiation to enhance the interaction between the incident radiation and the sample [37]. Our THz-TDS system exhibits an effective spectral range of 0.05–1.6 THz, with a signal-to-noise ratio of 10^5^ and a frequency resolution of 1.56 GHz.

### 2.3 Reagent

A series of lactose and amino acid solutions (lactose, cysteine, methionine, alanine, and phenylalanine dissolved in deionized water) with concentrations of 1–4 mg/ml are prepared to verify the sensing performance of the THz vernier sensor. In each experiment, a solution with a volume of 12 µl is dropped into Channel 3 with an effective sensing area of 24 mm^2^ and measured after drying. The weight percent of hydrogen peroxide solution used for oxidation is 0.005wt%, and the Ellman's Reagent used for the chromogenic reaction is 4 mg/ml (DTNB dissolving in 0.5 M Tris–HCl with pH = 8).

## 3 Results and discussion

### 3.1 Principle and design of THz vernier biosensor

The fundamental components and operational principles of the THz vernier sensor are illustrated in Figs. 1a and b. The multi-layer design establishes three transmission channels within the PPWG. Channel 1, located at the top layer, is filled with BOPP material exhibiting high transmittance in the frequency range of 0.1–1 THz. Channel 2 (the middle layer) consists of SiO_2,_ whose upper and lower surfaces are coated with gold films using magnetron sputtering technology to ensure strict spatial separation of the three channels within the waveguide. Finally, bottom Channel 3 is an air layer suitable for placing detected samples. Channel 2, serving as a sharing channel, is combined with Channel 1 and Channel 3 to construct overlapping MZI-1 and MZI-2. Due to the different FSRs of the two MZIs, resembling those of main and vernier scales in the vernier caliper, their interference spectra superimposed together generate the envelope. Following the principle of the vernier effect, the sensitivity and accuracy are significantly enhanced compared to MZI alone.

The schematic diagram of the THz vernier biosensor and its three transmission channels with relevant structural parameters are depicted in Fig. 1c. Herein, *l_m_* and *d_m_* denote the length and width (thickness) of the *mth* layer material. In the experiments, we employed BOPP material with *l*_1_ = 1.6 mm and *d*_1_ = 40 µm, SiO_2_ flakes with a length *l*_2_ of 6 mm and a thickness *d*_2_ of 130 µm, and the air layer of *d*_3_ = 30 µm. The tapered coupling angle for the PPWG is 10° [38]. Fig. 1d showcases photos capturing the SiO_2_ flakiness coated with gold films and the THz vernier biosensor utilized in our experiment. The transmission characteristics and sensing performance of the THz vernier biosensor are verified using a transmissive THz time-domain spectroscopy (THz-TDS) system in experiments shown in Fig. 1e, and system principle and parameters are illustrated in Methods.

**Fig. 2:**
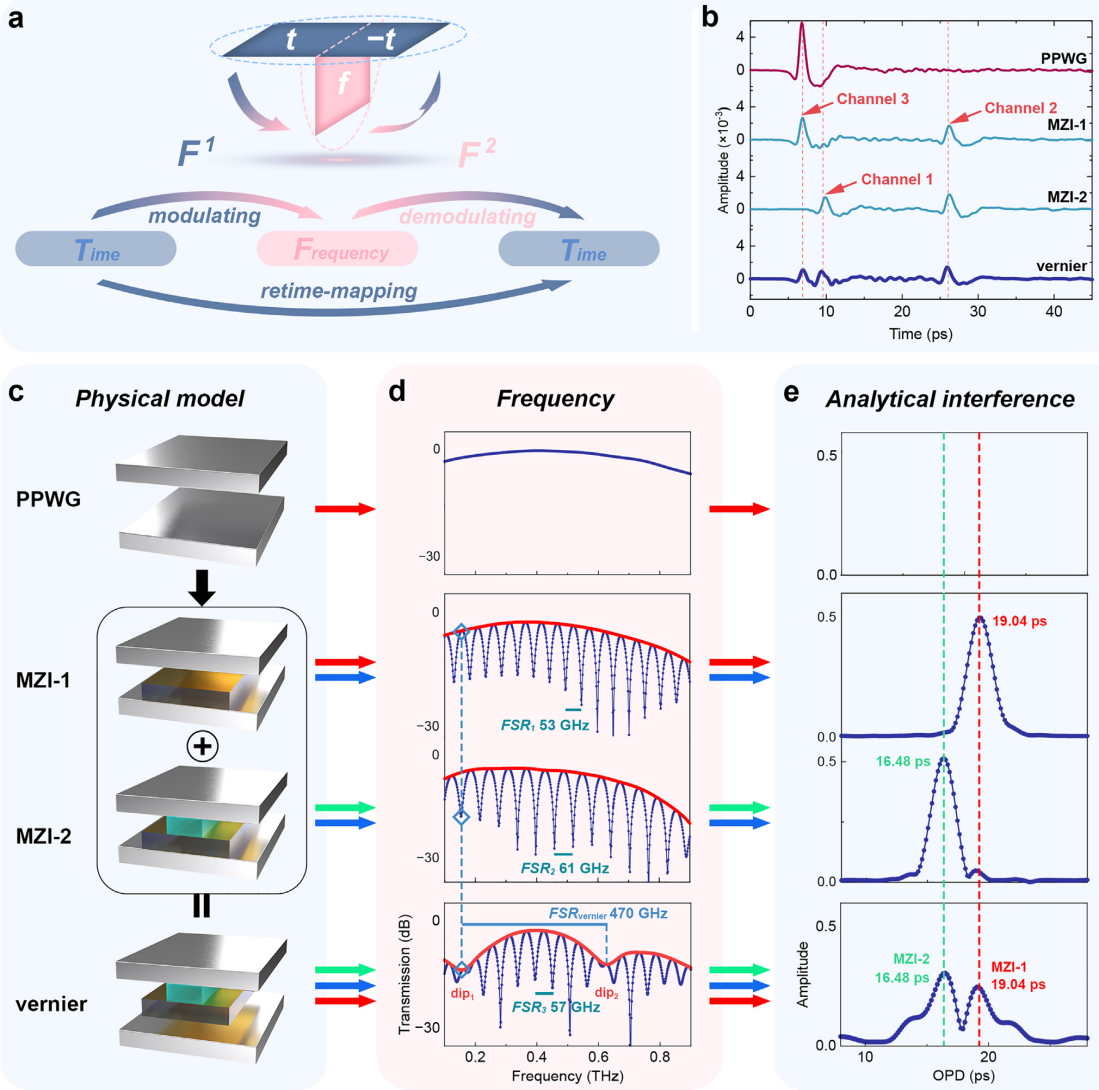
**The evolvement of THz vernier sensor.** (a) Diagram of the time-frequency-time mapping of the transmitted signal in the Fourier domain. *F*^1^, the first Fourier transform. *F*^2^, the second Fourier transform. (b) The time domain signal of the evolvement process of the THz vernier measured in the experiments. (c) Physical models shaping the THz vernier as the waveguide channels change. The (d) modulation and (e) demodulation for the analytical interference process in transmission spectra correspond to the waveguide channels. Red, blue, and green rows represent the THz radiation from different channels. Blue dashed lines indicate the dip of the enveloped spectrum. Green and blue sticks mean the different FSRs of MZI-1, MZI-2, and vernier. Green and red dashed lines mark the OPDs of the two MZIs. (For interpretation of the references to colour in this figure legend, the reader is referred to the web version of this article.)

The time-frequency-time mapping relationship in the Fourier domain is illustrated in Fig. 2a. The Fourier transform of the spectrum is utilized for detecting the interference mode, enabling the determination of both the magnitude and optical path difference of the interference. By performing the Fourier transform twice, coherent signals can be modulated and demodulated. In the demodulated spectrum, each peak corresponds to a specific interference mode, with the *x*-axis representing the optical path difference and the *y*-axis indicating coherence intensity. Following the Fourier inversion theorem, applying the Fourier transform twice will flip the time, which process is called retime-mapping.

Figs. 2b–e demonstrate the mapping characteristics of the THz vernier sensor signal in the Fourier domain. In the experiments, the evolvement process of the time domain signal in the THz vernier sensor is shown in Fig. 2b. The number of signal peaks indicates the number of channels without crosstalk. The constructions and the corresponding spectral modulation results are illustrated in Figs. 2c and d to illustrate the evolvement process shaping the THz vernier sensor, which can be dissected into four stages:

(1) The first stage depicts the standard PPWG mode in which the TM mode is characterized by negligible group velocity dispersion without a cutoff frequency and a single channel exhibiting high transmission across a wide frequency range [39]. Then, the spectrum is demodulated using the Fourier transform to acquire the analytical interferences, as shown in Fig. 2e, allowing for observation of interference modes, optical path difference (OPD) (Δ*nl*/*c*), coherence degree, and FSR in the time domain. Because of no interference in this physical model, the discernible characteristic curve cannot be observed in the demodulated spectrum.

(2) Second, dual channels establish the MZI-1 mode, with one channel comprising air and another containing SiO_2_ coated with gold film. The spectrum is modulated into periodic interference fringes with an *FSR*_1_ of 53 GHz. Demodulation analysis reveals a distinctive peak (red dashed line) near 19.04 ps, indicating OPD between the two channels. Remarkably, the reciprocal of the OPD is approximately 52.5 GHz, which is closely similar to the *FSR*_1_, showing the correspondence between the OPD and the FSR.

(3) In the third stage, the BOPP material replacing part of the air expands the optical path in the channel to construct the MZI-2 mode with the FSR modulated to 61 GHz and the OPD (green dashed line) reduced to 16.48 ps.

(4) In the final stage, the overlapping MZIs structure is formed by three channels to construct a THz vernier biosensor. Due to the similar FSRs of the two MZIs, their superimposed spectrum seems enveloped, where the dips (blue dashed lines) in the envelope are determined by the relative positions of that in MZIs. In the modulated spectrum, the *FSR*_3_ (57 GHz) of the interference dips is similar to that of each MZI. In comparison, the *FSR*_vernier_ (470 GHz) of the envelope shows significant improvement. At this point, characteristic peaks are observed in the demodulated spectrum at OPDs of 16.48 and 19.04 ps, respectively, confirming that the envelope spectrum originates from the combination of two MZI spectra.

### 3.2 Theoretical and optimal models for THz vernier biosensor

When two MZIs are overlapped, the superimposed interference spectrum seems enveloped since they have similar FSRs. The phase condition of the envelope is expressed as Eq. 1 [[40], [41], [42], [43]]:

(1) $$f_{\text{vernier}}=\frac{kc}{\Delta \delta },$$

where Δ*δ* = *δ*_1_ – *δ*_2_ means the OPD (Δ*nl*) between the two MZIs, *k* indicates the order of envelope dip, and *c* represents the speed of light in a vacuum. Differentiating the frequency of envelope dips enables sensitivity with the vernier effect to be expressed as Eq. 2, and the sensing accuracy (*A*_vernier_) can be calculated as Eq. 3:

(2) $$\begin{array}{c} S_{\text{vernier}}=\frac{f}{\Delta \delta }=\frac{f}{c}\frac{\mathrm{FS}R_{1}\cdot \mathrm{FS}R_{2}}{\mathrm{FS}R_{1}-\mathrm{FS}R_{2}}, \end{array}$$

(3) $$A_{\text{vernier}}=\frac{\Delta f}{S_{\text{vernier}}},$$

where *FSR_m_* means the FSR of MZI-*m*, and Δ*f* = 1.5625 GHz represents the spectrum resolution.

The magnification factor *M* of the THz vernier sensor can be approximately calculated as Eq. 4:

(4) $$M=\frac{S_{\text{vernier}}}{S_{\text{MZI}}}\approx \frac{\delta_{1}}{\Delta \delta }=\frac{FSR_{1}}{FSR_{1}-FSR_{2}}.$$

The detailed derivation for theoretical models of MZI and the THz vernier sensor can be seen in Section I in Supplementary I. Hence, the THz vernier sensing performance can be enhanced from two perspectives: firstly, reducing the Channel 1 length to achieve more similar OPDs in both MZIs will significantly improve sensitivity and magnification factor; secondly, according to effective medium approximations, the minor width of Channel 3 possesses the high volume proportion of sample within it, leading to a significant change in equivalent dielectric parameter of Channel 3 with the varying sample and thereby enhancing sensing sensitivity. The FDTD method is employed to validate these two approaches. Immense potential in sensing applications is demonstrated by numerical results that an optimally structural THz vernier sensor exhibits a sensitivity of up to 22.54 THz/RIU at operating frequencies near 0.9 THz, corresponding to an accuracy of 10^−5^ RIU (with a system resolution of approximately 1 GHz), which reports the highest performance as we know. Figs. S1–S3 in Supplementary I present a detailed process for optimizing the structure and showcasing the optimal sensitivity with ideal structural parameters. The results demonstrate that reducing the width of channel 3 leads to a significant increase in the effective volume ratio of the sample within channel 3, thereby enhancing sensing sensitivity. However, it is essential to decrease the widths of both channel 1 and channel 2 to maintain the desired splitting ratios in three channels and achieve optimal interference spectrum contrast. In our experiments, SiO_2_ with a minimum thickness of only 130 µm was utilized based on existing processes, which falls far short of the required thickness of 30 µm necessary for achieving a sensitivity of 22.54THz/RIU. Consequently, our experimental sensitivities were hindered by the thickness of SiO_2_.

### 3.3 Transmission characteristics of THz vernier biosensor

**Fig. 3:**
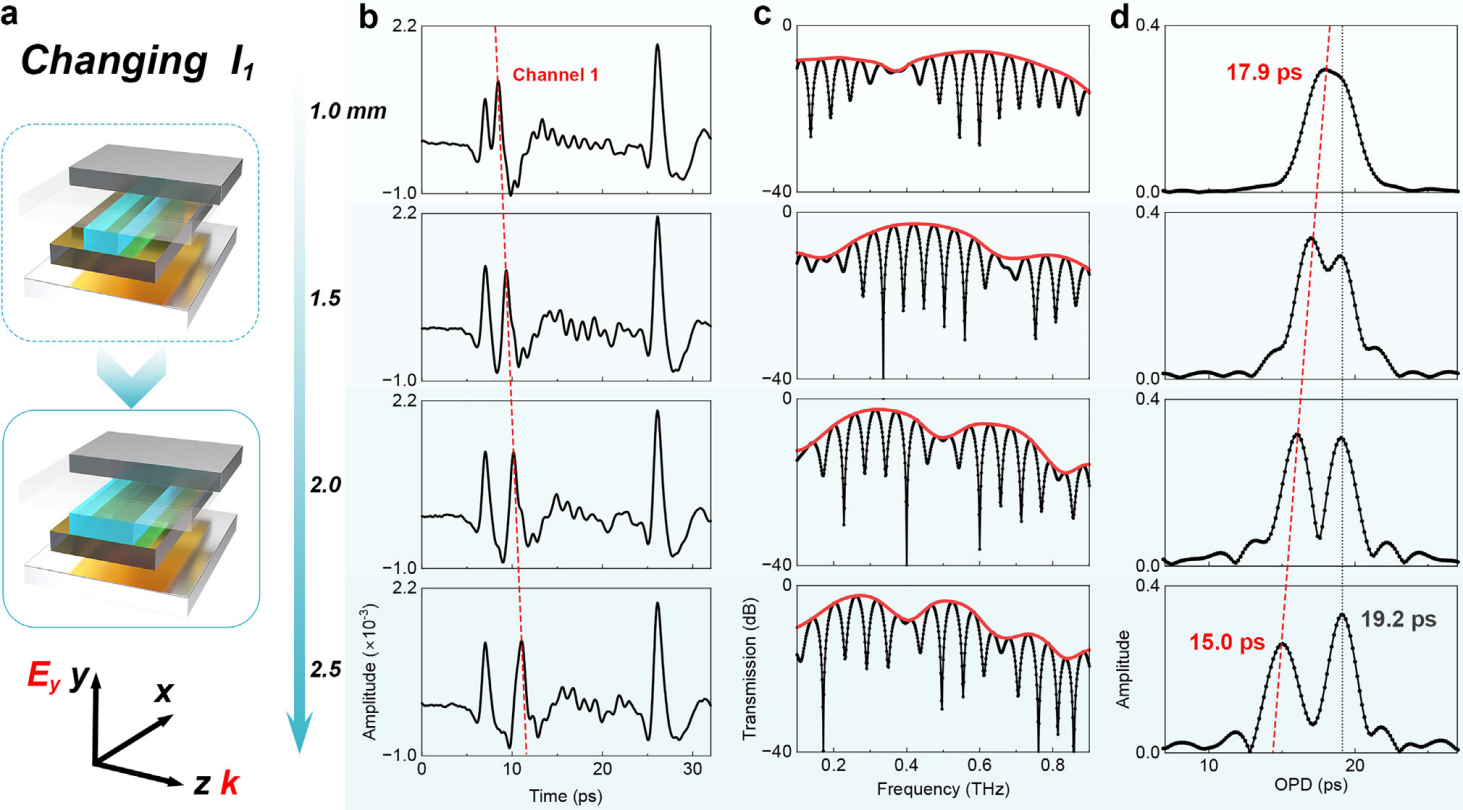
**Impact of Channel 1 length on transmission characteristics.** (a) Schematic diagram of BOPP length change in Channel 1. (b) The time domain signals varying with the length of Channel 1. (c) The modulation spectrum in the frequency domain obtained by once Fourier transform. (d) The demodulation spectrum in the time domain was obtained through twice Fourier transforms. The red solid line represents the envelopes of the spectra. The red dashed lines indicate the time signal in Channel 1 and the relevant OPD of MZI-2 in the demodulation spectrum. (For interpretation of the references to colour in this figure legend, the reader is referred to the web version of this article.)

The comprehensive evaluation of the THz vernier biosensor necessitates investigating the transmission characteristics. The impact of Channel 1 length (*l*_1_) on the transmission characteristics of the THz vernier is first studied, shown in Fig. 3. The impacts on the time domain signal are shown in Fig. 3b. As the length increases, the signal peak representing Channel 1 (red dashed line) needs a longer detection time, indicating the optical path in Channel 1 increases. However, the peaks of Channels 2 and 3 remain unchanged, meaning that no crosstalk occurs among these three channels within the THz vernier biosensor. The impact of *l*_1_ on the modulated spectrum is illustrated in Fig. 3c. With *l*_1_ increasing, a more significant number of envelope dips is observed. Because the optical path within Channel 1 extends, it leads to an augmented difference in FSRs between the two MZIs, consequently decreasing the FSR of the envelope. As depicted in Fig. 3d, the retime-mapping reveals a decrease in the OPD peak corresponding to MZI-2 from 17.9 ps to 15.0 ps, while the OPD peak representing MZI-1 remains unaltered at 19.2 ps. This result confirms that optical path change in Channel 1 only impacts MZI-2, and minor *l*_1_ is anticipated to achieve similar OPDs in two MZIs and boost sensitivity in experiments. Numerical simulation results (seen in Fig. S4 in Supplementary I) reached the same conclusion, verifying the reliability of the finding.

**Fig. 4:**
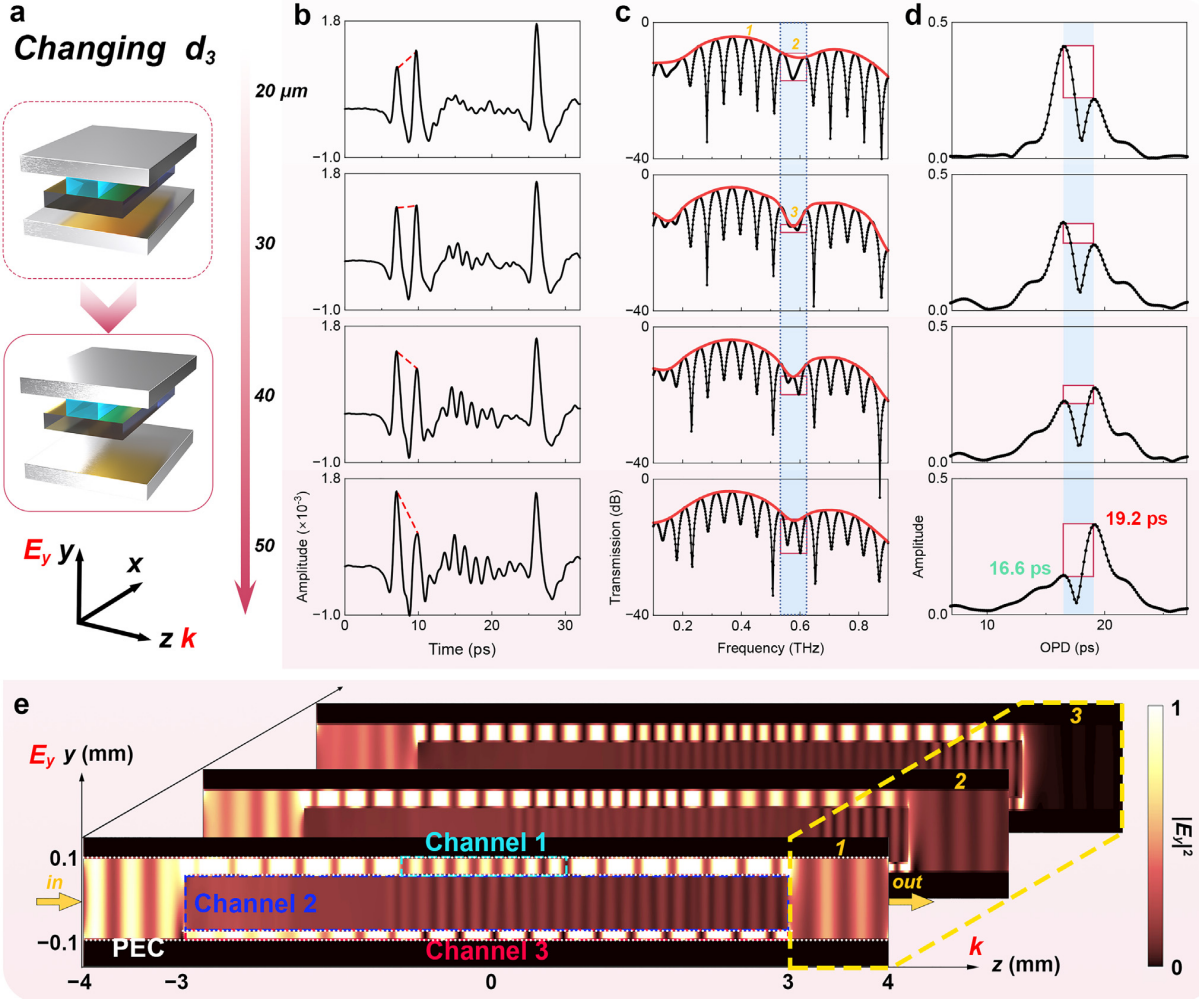
**Impact of Channel 3 width on transmission characteristics.** (a) Schematic diagram of width change in Channel 3. (b) The time domain signals varying with the width of Channel 3. (c) The modulation spectrum in the frequency domain obtained by once Fourier transform. (d) The demodulation spectrum in the time domain obtained through Fourier transforms twice. The blue rectangular areas represent the range of the envelope dips. The red rectangular areas indicate the contrast of the envelope dips. (e) The simulated electric field distribution at the position of the digital signs in (c). The white dotted lines mean the boundary condition of PEC. The yellow cuboid region includes the outport of the THz vernier sensor. (For interpretation of the references to colour in this figure legend, the reader is referred to the web version of this article.)

In addition to the attention devoted to the FSR of the THz vernier envelope, the investigation into the factor influencing the contrast of the envelope is also conducted, as depicted in Fig. 4a. In our structural design, Channel 2 serves as a shared channel for the formation of MZI-1 and MZI-2. By ensuring equal signal amplitudes in both Channel 1 and Channel 3, we achieve optimal contrast in the envelope spectrum. As a common pathway for both interferometers, Channel 2 has minimal impact on the contrast of the envelope spectrum. Therefore, our primary focus lies in investigating the effect of variations in the width (*d*_3_) of Channel 3 on the Channel 1 signal. The influence of *d*_3_ on the time domain signal can be observed in Fig. 4b. While the detection time of the three signals remains unchanged, variations in *d*_3_ significantly influence the amplitudes of signals in Channels 1 and 3. Specifically, an increase in *d*_3_ leads to an enhancing amplitude of the signal representing Channel 3, indicating a higher transmission through this channel. Correspondingly, the signal amplitude observed in Channel 1 gradually weakens. The impact of *d*_3_ on the modulation spectrum is illustrated in Fig. 4c. The blue rectangular region represents the range of envelope dips. It can be observed that variations in *d*_3_ will not modulate the frequency of these dips. However, there is a significant alteration in the contrast of the envelope dips, as indicated by the red rectangle areas, where similar energies in Channels 1 and 3 exhibit maximum contrast of the envelope dips. Conversely, an increase in energy disparity between these two channels leads to a substantial decrease in envelope contrast, as the comparison (*1*–*2* vs. *1*–*3*) in Fig. 4e shows. The retime-mapping depicted in Fig. 4d reveals that *d*_3_ impacts the coherence degree of MZIs while leaving the OPDs unaffected and a higher contrast of the envelope dips is observed when the amplitudes of the two OPDs are closer. The phenomenon above demonstrates that the variation in width primarily impacts the light-splitting ratio among channels while the optical path within these channels remains unchanged. A higher contrast is expected in experiments to improve precision. Relevant simulation results (seen in Fig. S5 in Supplementary I) are consistent with this conclusion. The research findings on the transmission characteristics of the THz vernier biosensor demonstrate that adjusting the structural parameters of the channels in the waveguide enables the modulation of the coherent signal.

### 3.4 Biosensing application with the THz vernier

A typical application of THz vernier is as a highly sensitive sensor. In sensing applications, only the transmission of MZI-1 is affected by the sample in Channel 3, whereas MZI-2 remains unaffected. The self-referencing property of the sensor arises from the reference effect of constant MZI-2 on MZI-1, where MZI-1 can be likened to a vernier scale in a vernier caliper and MZI-2 to the main scale. The difference frequency between these two interferometers, their superimposed spectra envelope, exhibits vernier gain whereby even a slight change in MZI-1 results in a significant alteration in their difference frequency envelope. Therefore, the self-referential THz vernier biosensor exhibits exceptional sensing sensitivity and accuracy by the gain of the vernier effect.

**Fig. 5:**
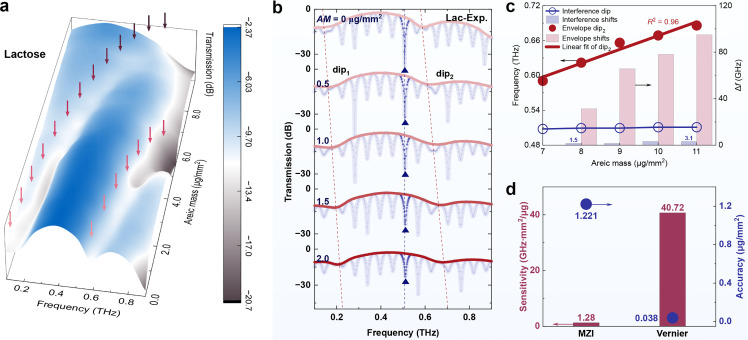
**Biochemical sample areic mass detection with THz vernier sensor.** (a) The envelope contour of THz vernier in detecting areic masses of lactose over broadband range. (b) Spectral characterization of lactose areic mass of 0–2 µg/mm^2^. The red gradient curves represent the envelopes, while the blue curves signify interference. The red and blue dashed lines correspond to envelope dips and interference dips, respectively. (c) The frequencies and shifts of interference and envelope dips in (b). (d) The lactose detection sensitivities and accuracies of interference and envelopes correspond to (b). AM, areic mass; Lac, lactose. (For interpretation of the references to colour in this figure legend, the reader is referred to the web version of this article.)

The capability for broadband sensing in the THz vernier biosensor is demonstrated by the detection of lactose with an areic masses range of 0–9 µg/mm^2^, shown in Fig. 5a. The envelope contour reveals that with an increase in lactose areic mass, envelope dip_2_ gradually exceeds the spectral range (>0.9 THz), while a continuous frequency shift of envelope dip_1_ is observed within the range of 0.15–0.8 THz. The compositive sensing span of these two dips exceeds 700 GHz, thereby confirming the broadband detection capability of the THz vernier sensor. The relevant detecting spectra can be seen in the GIF file in Supplementary II. It is worth noting that the discrepancy (Δ*δ*) in the OPDs between the two interferometers diminishes with an increasing sample, thereby indicating a discernible trend of heightened sensitivity, as demonstrated by Eq. 2. Nevertheless, when the sensor exhibits a narrow detection range, the linear approximation can still be employed for analyzing detection results. The THz vernier sensor response to different lactose areic masses is presented in Fig. 5b, where the two dips (red dashed lines) in envelopes (red gradient curves) exhibit a blue shift as the areic masses increase. This phenomenon can be attributed to an increase in the equivalent optical path in Channel 3, decreasing the OPD difference between the two MZIs, which aligns with Eq. 1 shown in Methods. Similarly, interference dips (blue dashed lines) within the spectrum (blue curves) also show a blueshift due to an increase in Channel 3 equivalent optical path and reduction of MZI-1 OPD.

The frequencies of the interference dip and envelope dip_2_ with their corresponding shifts are summarized in Fig. 5c. Notably, under vernier effect amplification, the frequency shift of the envelope dip is more significant than that of the interference dip. Sensitivities and accuracies for the two dip types are depicted in Fig. 5d. Experimental results reveal a high sensitivity of 40.72 GHz/(µg/mm^2^) and an accuracy of 0.038 µg/mm^2^ for envelope dips compared to only 1.28 GHz/(µg/mm^2^) and 1.221 µg/mm^2^ for interference dips. The self-referential THz vernier sensor achieves a remarkable sensitivity amplification of >3000% of a single THz MZI sensor. The sensing performance of a THz MZI sensor is demonstrated in Fig. S6 and Table S4. The high performance of the vernier sensor in detecting methionine and cysteine areic masses is also confirmed in Fig. S7. In addition, to further demonstrate the reliability of the THz vernier sensor, we conducted numerical simulations using both the thickness and the equivalent RI models in Fig. S8 in Supplementary I. The simulation results demonstrate an equivalent sensitivity of 570 GHz/RIU, with the vernier gain exceeding 3000% as well. These findings provide additional evidence for the exceptional performance of the self-referential THz vernier sensor.

**Fig. 6:**
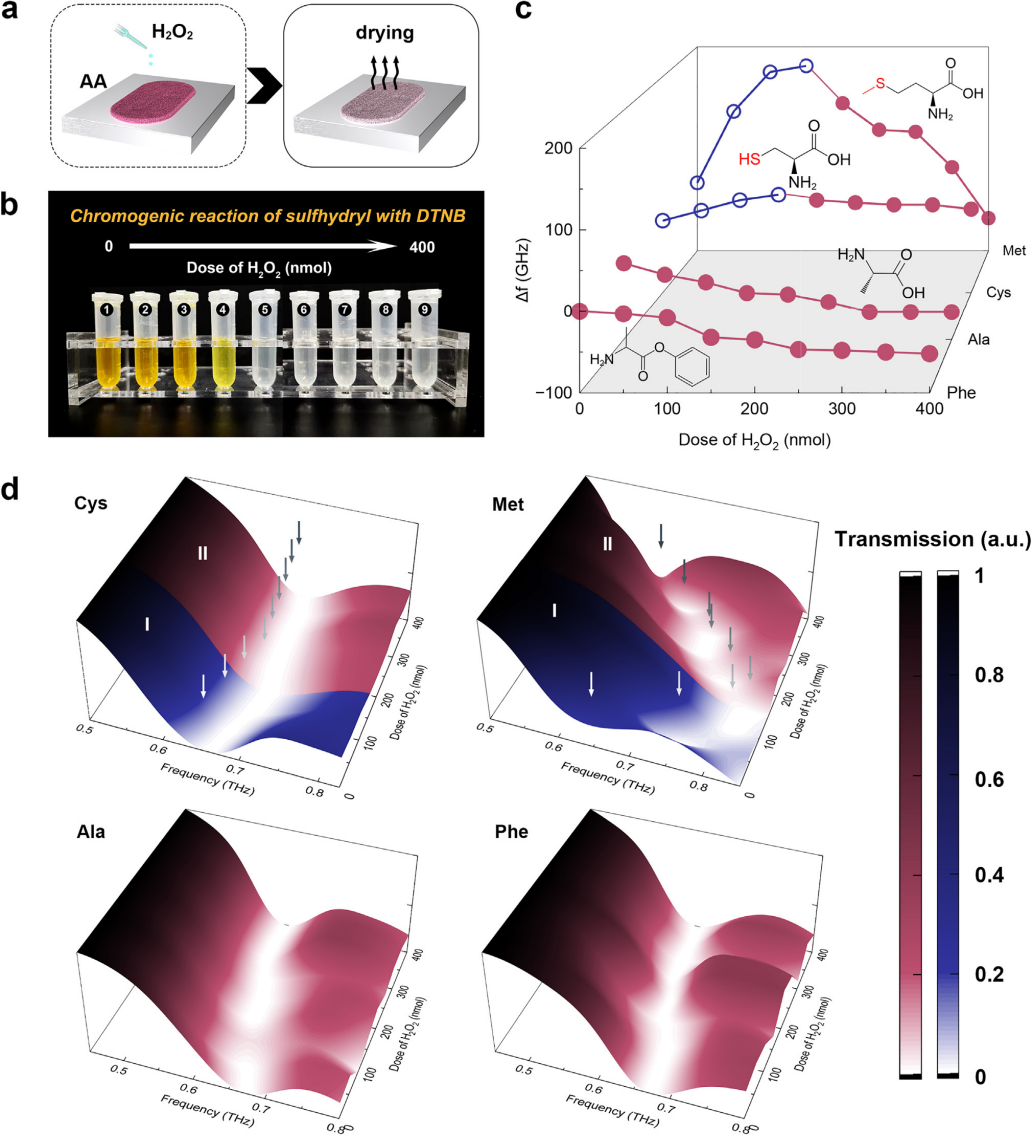
**Assistance in amino acid identification with THz vernier sensor.** (a) Schematic diagram of online detection of amino acid oxidized status. (b) The chromogenic reaction between DTNB and sulfhydryl groups in cysteine varying with the doses of hydrogen peroxide. (c) Characteristic curves of four amino acids (cysteine, methionine, alanine, and phenylalanine) reacting with different doses of hydrogen peroxide in the THz band. (d) The normalized envelope contour of the THz vernier sensor in detecting the four amino acids reacting with different doses of hydrogen peroxide. Blue and red dots indicate the blueshift and redshift, respectively. The arrows indicate the frequencies of envelope dips. DTNB, dithio-nitrobenzene. (For interpretation of the references to colour in this figure legend, the reader is referred to the web version of this article.)

The high sensitivity of the THz vernier sensor enables the detection of subtle changes in samples. We utilized this capability to monitor the oxidized status of amino acids. The schematic diagram of the real-time oxidation process of amino acids is shown in Fig. 6a. After drying, the normalized envelope contour obtained through the reaction between four amino acids and varying doses of hydrogen peroxide is illustrated in Fig. 6d. Taking cysteine as an example, the successive blueshifts (36 GHz) and redshifts (20 GHz) observed in the envelope contour indicate that as the doses of hydrogen peroxide increase from 0 to 400 nmol, cysteine undergoes a two-stage oxidation process [44]: In the first stage, with a low dose of hydrogen peroxide (<150 nmol), cysteine gradually transforms into cystine, leading to an increase in the equivalent optical path for Channel 3 and resulting in a blueshift in the envelope dip; In the second stage, with a high dose of hydrogen peroxide (>150 nmol), cysteine is oxidized into 3-Sulfoalanine causing a reduction in the equivalent optical path of Channel 3 and consequently inducing a red shift in the envelope dip. Chromogenic reactions between DTNB and sulfhydryl groups present in cysteine demonstrate the oxidized process above, as shown in Fig. 6b. Complete oxidation of cysteine is indicated by a color change from yellow to colorless within the mixed solution. However, discerning reaction processes from NO 1–3 and NO 5–9, based solely on color changes, proves challenging. By comparison, employing THz vernier sensors allows the determination of reaction endpoints and monitoring progress.

Furthermore, the characteristic curves of the reaction between four amino acids and hydrogen peroxide in the THz band are acquired, as illustrated in Fig. 6c. Similar to cysteine, methionine also undergoes two oxidation stages, forming methionine sulfoxide and methionine sulfone, respectively [45]. In contrast, amino acids without sulfur, selenium, or heterocycles, such as alanine and phenylalanine, typically exhibit stability in hydrogen peroxide. The redshift (about 50–60 GHz) of alanine and phenylalanine characteristic curves observed in Fig. 6d suggest their interaction with hydrogen peroxide, leading to their existence as perhydrates or co-crystals with hydrogen peroxide [46,47]. Exploring the reaction characteristic curves between amino acids and hydrogen peroxide enables monitoring of the oxidized status, which is determined by the molecular structure. It facilitates the identification of specific amino acid types based on significant differences in these curves. Successfully validating the sensing performance of THz vernier sensors in biochemical detection unveils novel prospects for THz photonics coherent detection.

## 4 Conclusion

We experimentally demonstrate the broadband THz vernier biosensor in the modulation and demodulation of coherent signals, showcasing its capability to continuously map the signal in the time-frequency-time domain where spectral characterization and analytical interference are performed. The self-referential characteristic of the THz vernier enables it to exhibit a 3000% sensitivity enhancement over the single interferometer in sensing. The areic mass detection of biochemical samples reaches 10^7^ GHz/(g/mm^2^), and the accuracy reaches 10^−8^ g/mm^2^. Additionally, we employ THz vernier sensors to characterize the amino acids characteristic curves in the THz band that exhibit significant differences determined by the molecular structure and allow the identification of amino acid species. Our work provides experimental demonstrations elucidating the mechanism of the THz vernier biosensor, opening new possibilities for future applications of THz photonics coherent technology in communications and biosensing.

## Declaration of competing interest

The authors declare that they have no conflicts of interest in this work.

## Appendix Supplementary materials

Image, application 1

Image, image 2
