# Supplementary material for: Retime-mapping terahertz vernier biosensor for boosting sensitivity based on self-reference waveguide interferometers
Source: Fundam Res. 2024 Dec 14;5(2):593–601. doi: 10.1016/j.fmre.2024.12.002 (PMC11997581; doi:10.1016/j.fmre.2024.12.002)
Supplement: Supplementary file 1 [file mmc1.pdf]

Supplementary Information I for

Retime-mapping terahertz vernier biosensor for boosting sensitivity based on self-reference waveguide interferometers

Liang Ma [a], Fei Fan [a,b,*], Weinan Shi [a], Yunyun Ji [a], Xianghui Wang [a], Shengjiang Chang [a,b,]**

[a] *Institute of Modern Optics, Nankai University, Tianjin Key Laboratory of Micro-scale Optical Information Science and Technology, Tianjin, 300350, China*
[b] *Tianjin Key Laboratory of Optoelectronic Sensor and Sensing Network Technology, Tianjin, 300350, China*
[*] *Corresponding author:* fanfei@nankai.edu.cn
[**] *Corresponding author:* sjchang@nankai.edu.cn

Contents

I. Theoretical model for THz vernier biosensor ....Page 2

II. Optimal structure for THz vernier biosensor ....Page 4

Figure S1 | Optimization of Channel 1 length .... Page 4

Table S1 | Sensitivities comparison with different Channel 1 length .... Page 4

Figure S2 | Optimization of Channel 3 width .... Page 5

Table S2| Sensitivities comparison with different Channel 3 width .... Page 5

Figure S3 | Optimal sensitivity with the ideal structure.................................. Page 6

Table S3 | Ideal parameters of THz vernier sensor. .... Page 6

III. THz vernier transmission characteristics in numerical simulation.........Page7

Figure S4 | Effects of Channel 1 length on transmission characteristics .... Page 7

Figure S5 | Effects of Channel 3 width on transmission characteristics .... Page 8

IV. THz vernier sensing performance verification..Page9

Figure S6 | Sensing performance in a single THz MZI .... Page 9

Table S4 | Comparison of sensing performance in THz vernier and MZI .... Page 9

Figure S7 | Areic mass detection of methionine and cysteine .... Page 10

Figure S8 | THz vernier sensitivity characterized by thickness and equivalent refractive index models .... Page 11

I. Theoretical model for THz vernier biosensor

The modulation of spectral intensity by a single MZI-*m* satisfies Eq. (S1):

$$I_{\text{MZI-1(2)}} = I_{C_2} + I_{C_{3(1)}} + 2\sqrt{I_{C_2} I_{C_{3(1)}}} \cos\left(\frac{2\pi f \delta_{1(2)}}{\text{c}}\right), \tag{S1}$$

where I_m denotes the terahertz (THz) radiation intensity, C_m is the number of channels, δ_m means the optical path difference (OPD) (Δnl) of two channels in MZI-*m*, and c represents the speed of light in vacuum. Hence, the phase condition of interference is expressed as Eq. (S2):

$$f_{\text{MZI-}m} = \frac{k\text{c}}{\delta_m}, \tag{S2}$$

where $f_{MZI\text{-}m}$ means the interference dip of MZI-*m*, and *k* indicates the order of interference frequency. By differentiating the interference frequency, the free spectral range (FSR) of MZI-*m* can be expressed as Eq. (S3):

$$FSR_{\text{MZI-}m} = \frac{\text{c}}{\delta_m}, \tag{S3}$$

from which, the FSR and OPD are considered equivalent, with their product consistently equal to the speed of light. Hence, by differentiating the interference frequency, the two expressions of sensitivity can be described as Eqs. (S4) and (S5):

$$S_{\text{MZI-}m} = \frac{f}{\delta_m}, \tag{S4}$$

$$S_{\text{MZI-}m} = \frac{f}{\text{c}} FSR_m. \tag{S5}$$

When two MZIs are overlapped, the superimposed interference spectrum seems enveloped since they have similar FSRs. Hence, the total intensity and the frequencies of envelope dips are modulated as Eq. (S6):

$$I_{\text{nonius}} = \sum I_{\text{MZI-}m} = I_{C_1} + I_{C_2'} + I_{C_2''} + I_{C_3} + 2\sqrt{I_{C_2'} I_{C_3}} \cos\left(\frac{2\pi f \delta_1}{\text{c}}\right) + 2\sqrt{I_{C_2''} I_{C_1}} \cos\left(\frac{2\pi f \delta_2}{\text{c}}\right), \tag{S6}$$

where $I_{C'2} + I_{C''2} = I_{C2}$. Assuming that $I_{c_1} = I_{c_3}$ and $I_{c'_2} = I_{c''_2}$, the total intensity can be simplified as Eq. (S7):

$$I_{\text{nonius}} = A + B\cos\left(C(\delta_1 + \delta_2)\right)\cos\left(C(\delta_1 - \delta_2)\right), \tag{S7}$$

where $A = 2(I_{c_1} + I_{c_3})$, $B = 2\sqrt{(I_1 I_3)}$, and $C = 2\pi f / \text{c}$. In Eq. (S7), the high-frequency components of the two cosine terms represent the interference spectrum, whereas the low-frequency components correspond to the envelope spectrum. Hence, the phase condition of the envelope is expressed as Eq. (S8):

$$f_{\text{nonius}} = \frac{k\text{c}}{\Delta\delta}, \tag{S8}$$

where $\Delta\delta = \delta_1 - \delta_2$ means the OPD difference between the two MZIs. Differentiating the frequency of envelope dips enables the FSR and sensitivity with the vernier effect to be expressed as Eq. (S9):

$$FSR_{\text{nonius}} = \frac{\text{c}}{\Delta\delta}, \tag{S9}$$

from which the FSR_{vernier} and OPD differences in the two MZIs are considered equivalent. Hence, by differentiating the interference frequency, the two expressions of envelope sensitivity

can be described as Eqs. (S10) and (S11):

$$S_{\text{nonius}} = \frac{f}{\Delta\delta}, \tag{S10}$$

$$S_{\text{nonius}} = \frac{f}{\text{c}} \frac{FSR_1 \cdot FSR_2}{FSR_1 - FSR_2}. \tag{S11}$$

When $\delta_1 \approx \delta_2$ or $FSR_1 \approx FSR_2$, the magnification factor M of the THz vernier can be approximately calculated as Eq. (S12):

$$M = \frac{S_{\text{nonius}}}{S_{\text{MZI}}} \approx \frac{\delta_1}{\Delta\delta} = \frac{FSR_1}{FSR_1 - FSR_2}, \tag{S12}$$

from which similar OPDs or FSRs of two MZIs will significantly improve the sensitivity in the vernier sensor.

II. Optimal structure for THz vernier biosensor

The sensitivity of the THz vernier sensor was calculated using the finite difference time domain method, as illustrated in Figure S1. By reducing the Channel 1 length, a significant enhancement in the response of the envelope dip to the sample was observed. For instance, at l_1 = 1.4 mm, a response within a frequency range of 323 GHz was detected; however, only a response of 108 GHz was observed at l_1 = 2.0 mm. The demodulation results indicate that Channel 1 length only influences the OPD of MZI-2, while a higher sensitivity is achieved when both MZIs have similar OPDs. The frequency shift and sensitivity curves demonstrate a threefold increase in sensitivity when reducing Channel 1 length from 2 mm to 1.4 mm.

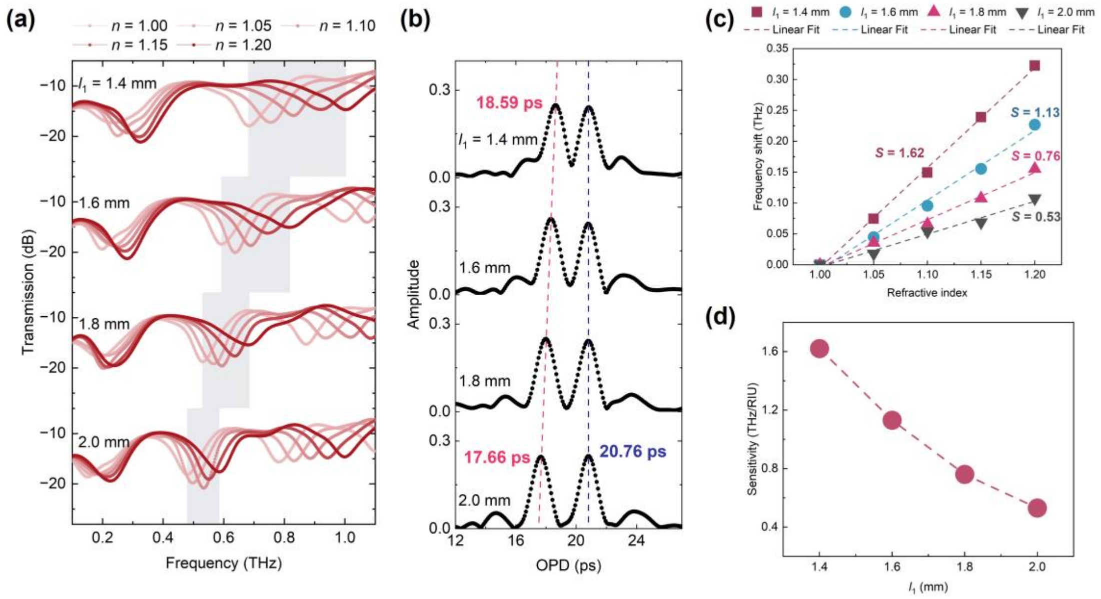

Figure S1 Optimization of Channel 1 length. (a) Envelope dip sensitivity varying with Channel 1 length. The gray backgrounds indicate the response range of the envelope dips. (b) Coherent signal demodulation results changing with the Channel 1 length without sample. Red and Blue dashed lines indicate the changing OPDs in MZI-2 and MZI-1, respectively. (c) Frequency shifts of envelope dips in (a). (d) The sensitivities in **c** as a function of Channel 1 length.

When the Channel 1 length is increased, it leads to a corresponding change in the mid-frequency of the selected envelope dip. Consequently, to mitigate the influence of frequency, we employ the ratio of sensitivity to mid-frequency. The same conclusion is demonstrated that reducing Channel 1 length can enhance sensitivity, as shown in Table S1.

Table S1 The comparison of sensitivities with and without mid-frequency influence.

Channel 1 length l_1 (mm)	Sensitivity S (THz/RIU)	Mid-frequency f_0 (THz)	S / f_0 (RIU^{-1})
**1.4**	**1.62**	0.843	**1.922**
1.6	1.13	0.706	1.601
1.8	0.76	0.607	1.252
2.0	0.53	0.532	0.996

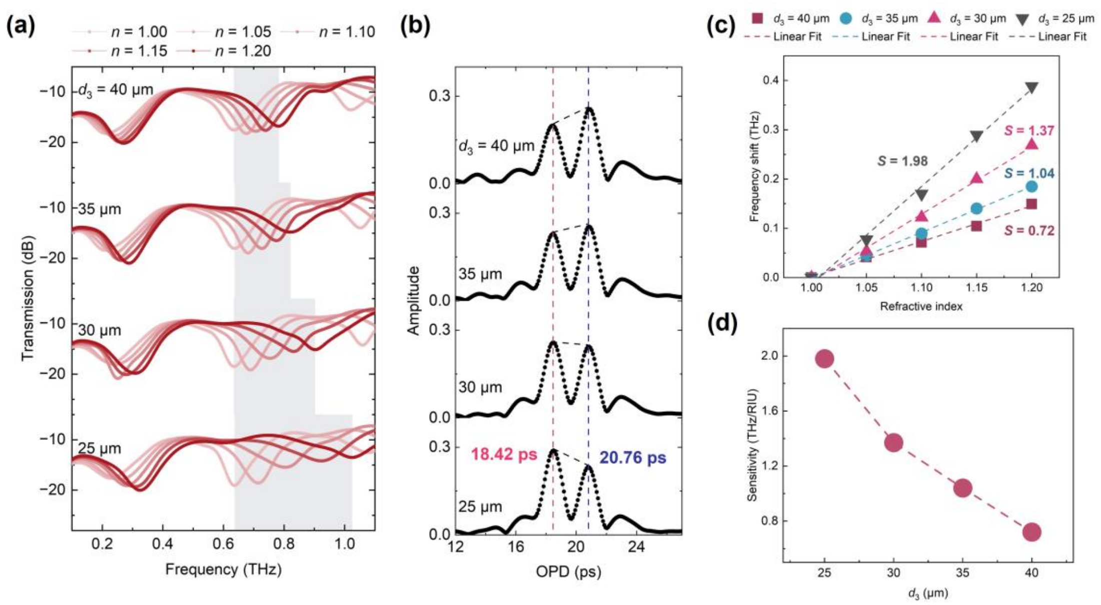

Figure S2 Optimization of Channel 3 width. (a) Envelope dip sensitivity changing with Channel 3 width. The gray backgrounds indicate the response range of the envelope dips. (b) Coherent signal demodulation results varying with the Channel 3 width without sample. Red and Blue dashed lines indicate the changing OPDs in MZI-2 and MZI-1, respectively. (c) Frequency shifts of envelope dips in (a). (d) The sensitivities in **c** as a function of Channel 3 width.

In addition to utilizing similar OPDs of the two MZIs, an effective approach to enhance sensitivity has been validated, namely, diminishing the Channel 3 width to augment the sample volume ratio within this channel, as shown in Figure S2. The envelope dip response to the sample increases from 149 GHz to 388 GHz as Channel 3 width decreases from 40 μm to 25 μm. The demodulation results demonstrate that altering Channel 3 width only impacts the splitting ratio within channels without affecting the OPDs of the two MZIs. The frequency shift and sensitivity curves visually indicate that reducing Channel 3 width is advantageous for enhancing THz vernier sensitivity. The sensitivity results are summed up in Table S2.

Table S2 The comparison of sensitivities with different Channel 3 widths.

Channel 3 width $\boldsymbol{d_3}$ (μm)	Sensitivity $\boldsymbol{S}$ (THz/RIU)
40	0.72
35	1.04
30	1.37
**25**	**1.98**

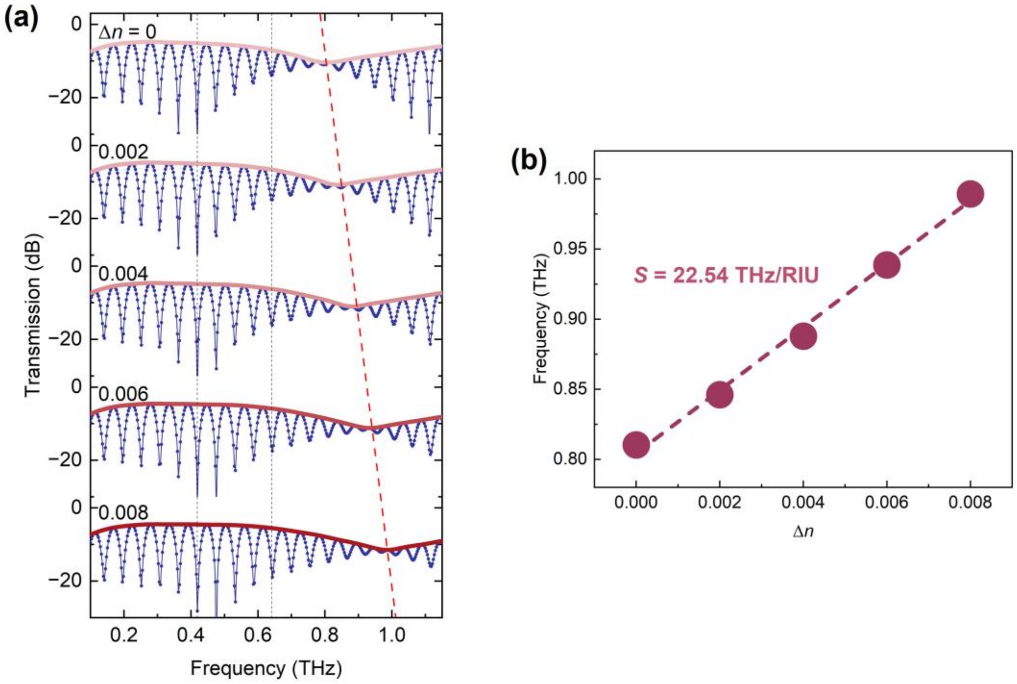

Figure S3 Optimal sensitivity with the ideal structure. (a) The spectrum response to the sample refractive index in a thousandth magnitude with ideal THz vernier sensor structural parameters. (b) Sensitivity linear fitting result in (a).

The above numerical results confirm that the sensitivity of THz vernier can be significantly enhanced by optimizing structural parameters. Optimal structural parameters were employed to achieve the highest sensitivity, as depicted in Figure S3. The envelope dip exhibits a response to sample refractive index changes of one thousandth magnitude (the red dashed line), which is challenging to detect using the interference dip (the black dashed line). This result demonstrates the capability of the THz vernier sensor to amplify interferometer sensitivity. Linear fitting analysis reveals an astonishingly high sensitivity value of 22.54 THz/RIU for the THz vernier sensor with ideal structural parameters, highlighting its immense potential in sensing applications. The key structural parameters are presented in Table S3. However, due to challenges in the material dimension, the structural parameters used in experiments deviate from the ideals.

Table S3 The ideal structural parameters of THz vernier sensor.

	length (mm)	width (μm)
Channel 1	**1**	10
Channel 2	6	30
Channel 3	-	**7**
sample	6	5

III. THz vernier transmission characteristics in numerical simulation.

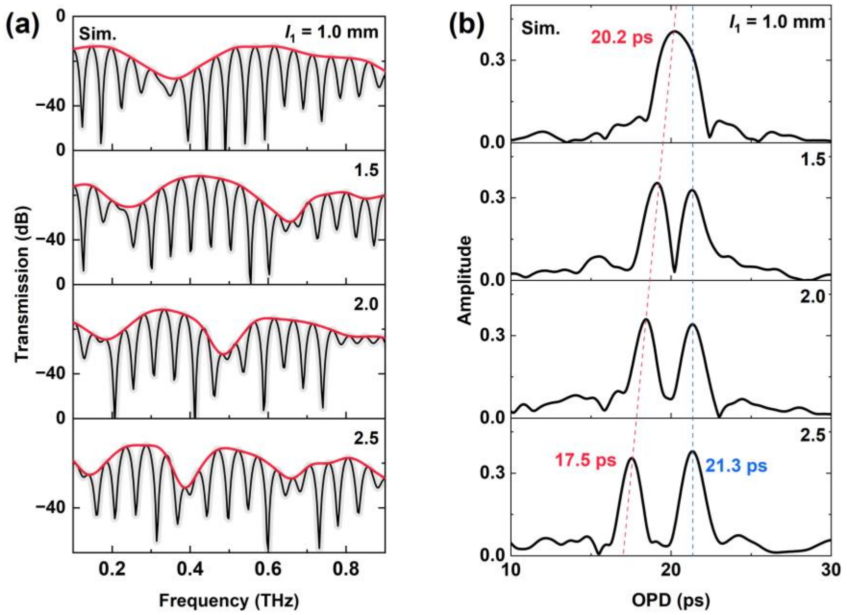

Figure S4 Effects of Channel 1 length on transmission characteristics. The numerical simulation results of the THz vernier biosensor varying with the length of Channel 1 in the (a) frequency domain and (b) demodulation spectra in the time domain. Red solid lines represent the envelopes of spectra. The red and blue dashed lines indicate the OPD peaks of MZI-2 and MZI-1, respectively.

Here, we employed the *finite difference time domain* method to investigate the impact of BOPP material length in Channel 1 on the THz vernier biosensor, as shown in Figure S4. The simulation assumed a refractive index of n = 2.05 and an extinction coefficient of κ = 0.005 for SiO_2, while n = 1.5 and κ = 0.005 were considered for BOPP. Increasing the length of BOPP from 1 mm to 2.5 mm decreased the free spectral range (FSR) of the envelope spectrum, and more envelope dips can be observed within the effective spectrum range. Spectral interference analysis revealed that the optical path difference (OPD) peak representing Mach-Zehnder interferometer-2 (MZI-2) gradually decreased from 20.2 ps to 17.5 ps. In contrast, the OPD peak representing MZI-1 remained unchanged at 21.3 ps, indicating that increasing BOPP length only affected the OPD of MZI-2 without influencing the OPD stability of MZI-1. By adjusting the OPD of MZI-2, it is possible to modulate both the frequency of envelope dips and the FSR of the envelope in the spectrum.

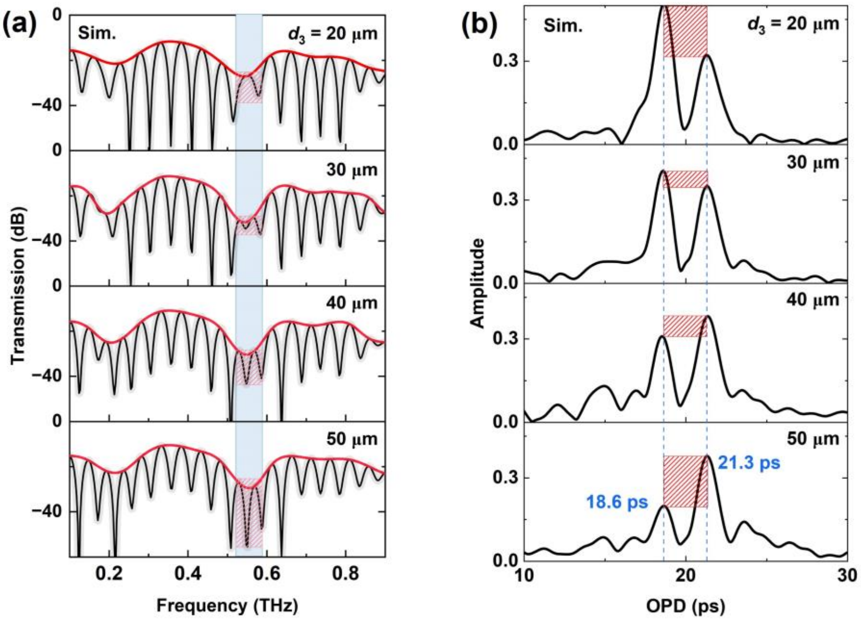

Figure S5 Effects of Channel 3 width on transmission characteristics. The numerical simulation results of the THz vernier sensor varying with the width of Channel 3 in the (a) frequency domain and (b) demodulation spectra in the time domain. Red solid lines represent the envelopes of spectra. The blue rectangle area indicates the envelope dips frequency range. The blue dashed lines mean the unchanged OPD peaks of MZI-2 and MZI-1. Red diagonal line areas mean the envelope contrast.

In addition to investigating the impact of Channel 1 length, we also examined the effect of Channel 3 width on the THz vernier spectrum, as depicted in Figure S5. The contrast of the envelope spectrum varies with changes in Channel 3 width. When Channel 3 is 30 μm wide, THz wave intensities in Channels 1 and 3 are comparable, resulting in a similar interference contrast in MZI-1 and MZI-2. For other widths, there is a significant difference between intensity in Channels 1 and 3, leading to reduced envelope contrast. This conclusion was confirmed by interference demodulation spectra analysis. High envelope contrast corresponds to similar OPD peaks for both interferometers, indicating that energy is similar across channels. In contrast, decreased envelope contrast reflects more significant differences between OPD peaks, suggesting increased energy disparity between Channels 1 and 3. Our findings demonstrate that Channel 3 width primarily affects channel splitting ratios and modulates envelope contrast.

IV. THz vernier sensing performance verification

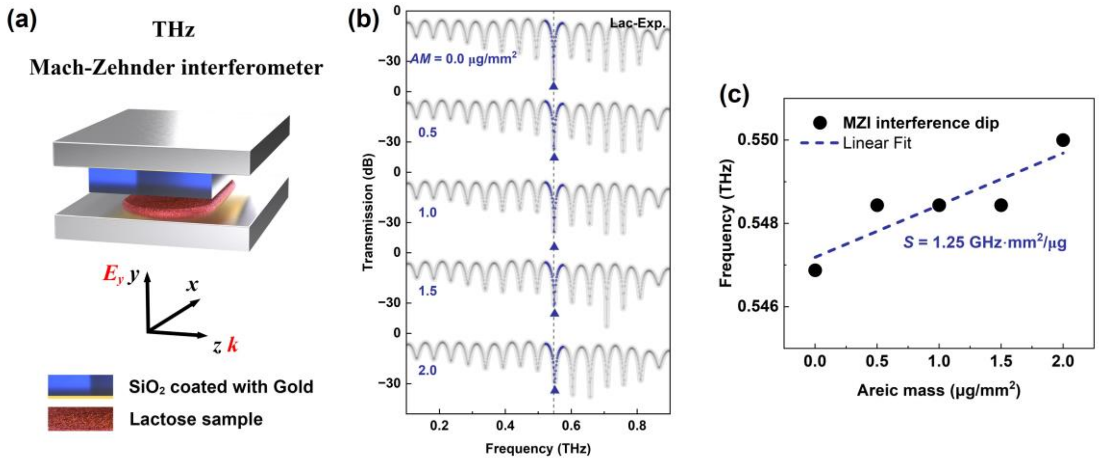

Figure S6 Sensing performance in a single THz MZI. (a) Schematic diagram of a THz Mach-Zehnder interferometer sensor based on a metallic dual-channel parallel-plate waveguide. (b) Spectral characterization of lactose areic mass of 0–2 μg/mm^2 measured by the THz MZI sensor in (a). (c) The frequencies of interference dips in (b) and the linear fit sensitivity of the THz MZI sensor. Lac, lactose; AM, areic mass; MZI, Mach-Zehnder interferometer. The black dashed line is utilized to facilitate the comparison of the frequency shift of interference dip around 0.54 THz.

Table S4 Comparison of sensing performance.

	**Interference dip in THz MZI**	**Interference dip in THz vernier**	**Envelope dip in THz vernier**
**Sensitivity (GHz·mm²/μg)**	1.25	1.28	**40.72**
**Accuracy (μg/mm²)**	1.25	1.221	**3.84×10^{-2}**

[a] Accuracy is calculated by Δ/S, where S means the sensitivity, and Δ represents the frequency resolution of the THz-TDS system that $\Delta = 1.5625$ GHz.

The THz MZI sensor, as depicted in Figure S6(a), was fabricated, and the enhanced sensitivity of the THz vernier sensor was validated. The structural parameters of the THz MZI sensor closely resemble those of the THz vernier sensor described in the article. Specifically, a gold film is coated on the SiO_2 surface to construct a dual-channel configuration in a metallic PPWG. One channel serves as the reference arm and is filled with SiO_2, having a thickness of 130 μm along the y-axis and a transmission length of 6 mm along the z-axis. The other channel functions as a sensing arm for sample placement and consists of an air gap with a thickness of 30 μm along the y-axis. During experimentation, 1–4 mg/ml of lactose solutions were prepared using deionized water as the solvent, with a concentration gradient of 1 mg/ml. The spectral response of the THz MZI sensor to lactose areic mass is illustrated in Figure S6(b). As the lactose areic mass increases from 0 to 2 μg/mm^2, a frequency shift of approximately 3 GHz is observed in the interference dip near 0.54 THz. The detection sensitivity of lactose by the THz MZI sensor

is depicted in Figure S6(c). A sensitivity value of 1.25 GHz·mm^2/μg was obtained by fitting the interference dips. Table S4 compares sensing performance between the interference dip of THz MZI, the interference dip of THz vernier, and the envelope dip of THz vernier. It can be observed that both the interference dip of THz MZI and THz vernier exhibit similar levels of sensitivity and accuracy. Moreover, under the gain of the vernier effect, the envelope dip achieved more than a 30 times enhancement in sensitivity and accuracy.

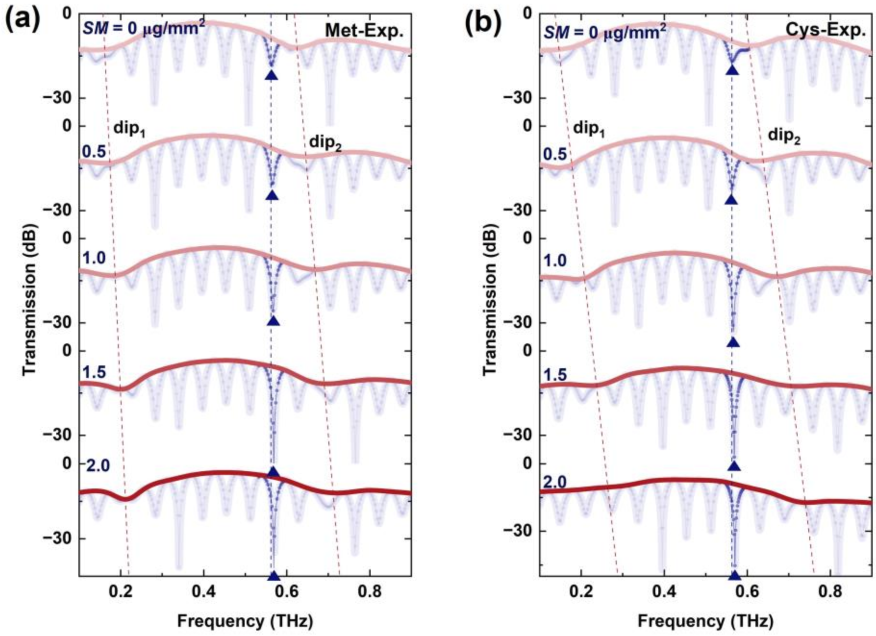

Figure S7 Areic mass detection of methionine and cysteine. Spectral characterization of (a) methionine and (b) cysteine areic mass detection. The blue dashed line assists in observing the interference spectrum frequency shift. Red dashed lines indicate the envelope dips change with the areic mass of methionine. AM, areic mass; Met, methionine.

To validate the sensing performance and sensitivity enhancement of the THz vernier sensor, we also conducted measurements on the areic mass of methionine and cysteine, as depicted in Figure S7. The concentrations of methionine and cysteine were 1, 2, 3, and 4 mg/ml. As shown in Figure S7(a), with an increase in methionine areic mass from 0 to 2.0 μg/mm^2, the interference spectrum demonstrates a blueshift of approximately 4 GHz, while the envelope spectrum exhibits a blueshift of around 90 GHz, resulting in a sensitivity enhancement exceeding twentyfold. Figure S7(b) presents similar results.

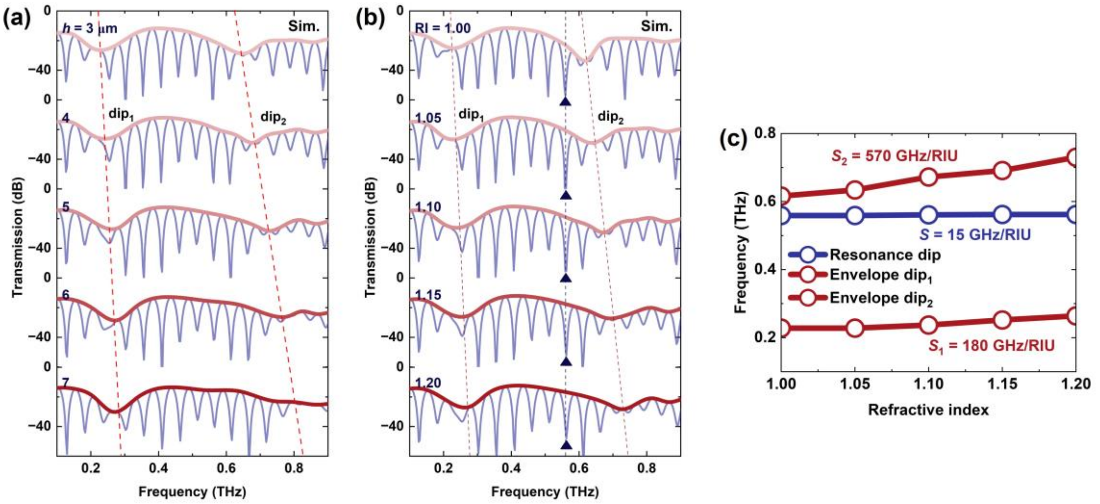

Figure S8 THz vernier sensitivity characterized by thickness and equivalent refractive index models. (a) Thickness model and (b) Equivalent refractive index model characterizing the sensitivity of THz vernier sensors. (c) Numerical calculations of sensitivities in equivalent refractive index model. The blue dashed line assists in observing the interference spectrum frequency shift. Red dashed lines indicate the envelope dips change with the sample thickness and refractive index. RI, refractive index.

To validate the sensing performance and sensitivity enhancement of the THz vernier sensor, we employed a thickness model and an equivalent refractive index model to simulate the sensing process, as depicted in Figure S8. In the thickness model, with a fixed sample refractive index of 1.3, increasing the sample thickness from 3 μm to 7 μm resulted in both blueshifts in the interference spectrum and envelope. Notably, the frequency shift of the envelope was more pronounced, as seen in Figure S8(a). We devised an equivalent refractive index model with a constant sample thickness of 5 μm to assess sensor sensitivity. As the equivalent refractive index increased from 1 to 1.2, approximately a 3 GHz frequency shift was observed in the interference spectrum, while about a 114 GHz frequency shift occurred in envelope dip_2, as shown in Figure S8(b). The calculated results for sensitivity are presented in Figure S8(c). Specifically, our findings indicate that while the interference spectrum exhibited a sensitivity of 15 GHz/RIU, envelope dip_2 demonstrated a significantly higher sensitivity of about 570 GHz/RIU, approximately 38 times greater than that observed for the interference spectrum. Simulation and experimental outcomes collectively confirm that the THz vernier sensor exhibits exceptional detection sensitivity and significant sensitivity enhancement.
