# Supplementary figures and images for: Retime-mapping terahertz vernier biosensor for boosting sensitivity based on self-reference waveguide interferometers
Source: Fundam Res. 2024 Dec 14;5(2):593–601. doi: 10.1016/j.fmre.2024.12.002 (PMC11997581; doi:10.1016/j.fmre.2024.12.002)

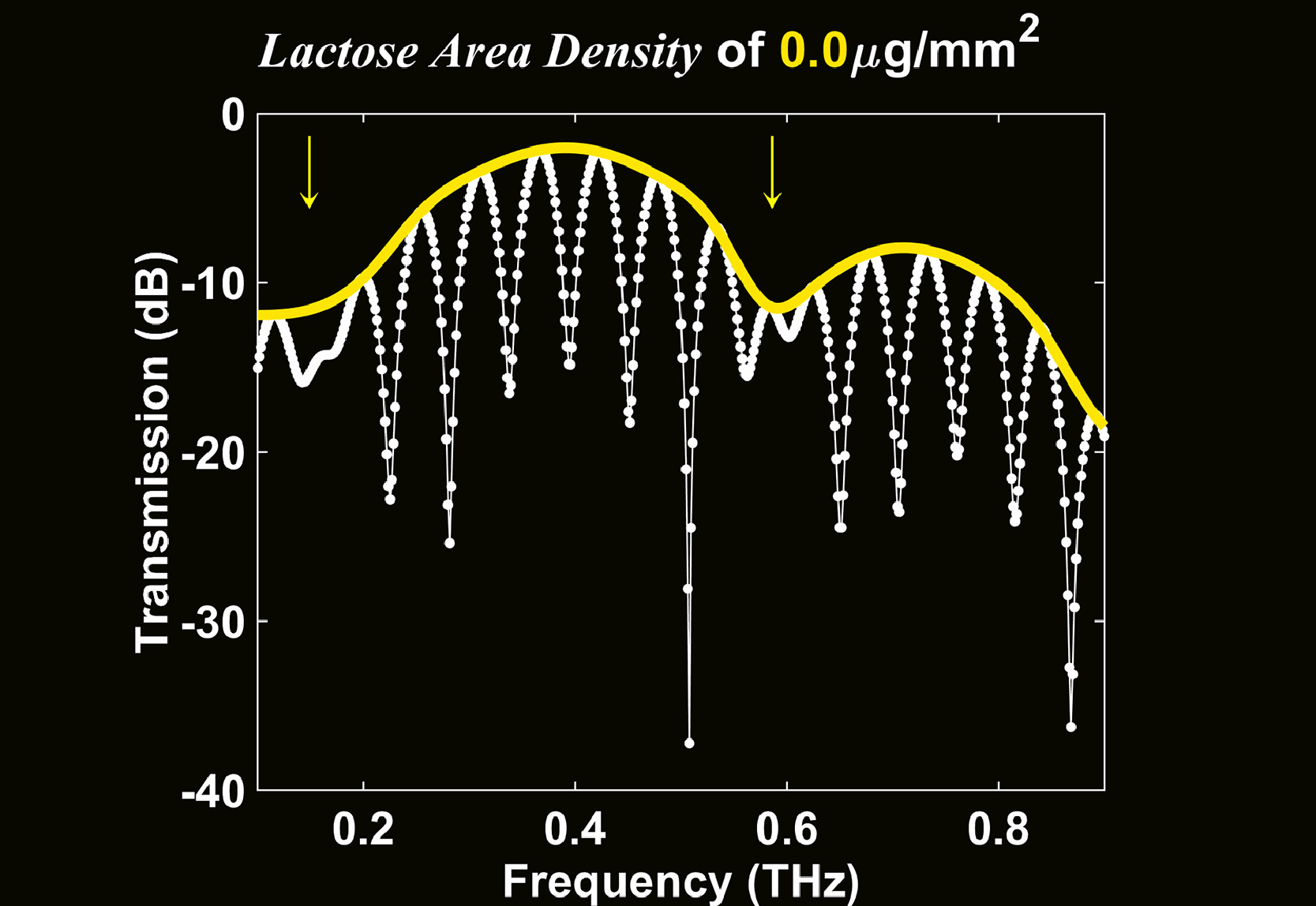

Supplement: Supplementary file 2 [file mmc2.jpg]
